# TL1-A can engage death receptor-3 and activate NF-kappa B in endothelial cells

**DOI:** 10.1186/1471-2369-15-178

**Published:** 2014-11-16

**Authors:** Jun Wang, Rafia S Al-Lamki, Xinwang Zhu, Hanzhe Liu, Jordan S Pober, John R Bradley

**Affiliations:** Department of nephrology, First Hospital of China Medical University, Nanjing Street, 110001 Shenyang, P.R. China; Department of Medicine, University of Cambridge, School of Clinical Medicine, Addenbrooke’s Hospital, Box 157, Hills Road, Cambridge, CB2 0QQ UK; The Boyer Centre for Molecular Medicine, Yale University School of Medicine, 10 Amistad Street, Room 401D, New Haven, CT 06520 USA

**Keywords:** Kidney, Endothelial cells, Death receptor, Inflammation

## Abstract

**Background:**

Death receptors (DRs) play an important role in renal pathology. We have shown that DR3 is inducibly expressed on renal tubular epithelial cells in the setting of inflammatory injuries. In this study we investigate the expression of DR3 in renal endothelial cells and their response to TL1A, the only known ligand of DR3.

**Methods:**

We did RT-PCR, flow cytometry and subcellular immunoblotting to examine the expression and function of DR3 in cells in vitro. We did organ culture of human and mouse tissue to examine expression and signal of DR3 in vivo.

**Results:**

DR3 is expressed in some interstitial vascular endothelial cells (EC) in human kidney in situ; these EC also respond to its ligand TL1A by activating NF-κB. Very low levels of DR3 can be detected on the cell surface of cultured human umbilical vein (HUV) EC, which do not respond to TL1A. HUVEC transfected to overexpress DR3 become responsive to TL1A, assessed by IκBα degradation and E-selectin induction, indicating that the signaling components needed for DR3 responsiveness are expressed. TL1A induces NF-κB activation in EC in renal and cardiac tissue from wild type but not DR3 knock-out mice.

**Conclusion:**

TL1A and DR3 activate NF-κB in vascular endothelial cells, and can be an important regulator of renal interstitial vascular injury.

**Electronic supplementary material:**

The online version of this article (doi:10.1186/1471-2369-15-178) contains supplementary material, which is available to authorized users.

## Background

Death receptors (DRs) of the tumor necrosis factor receptor (TNFR) superfamily include TNFR1, Fas, DR3, DR4, DR5 and DR6. The members of DR family each contain an intracellular death domain (DD) that binds to adaptor proteins which also contain DDs and initiate apoptotic cell death [1]. The DD of DR3 shares the highest homology with that of TNFR1 (47%) [2], and, like TNFR1, interacts with the DD-containing silencer of death domain (SODD) in the absence of ligand, whereas the other four DRs do not [3, 4]. Furthermore, upon ligand binding, only DR3 and TNFR1 recruit the adaptor TNFR-associated death domain (TRADD) protein and assemble a TRADD-dependent signalling complex that rapidly triggers NF-κB and c-Jun N-terminal kinase (JNK) activation; after a delay of several hours the TRADD signalosome recruits Fas-associated DD (FADD) protein that may trigger apoptosis [5]. However, in contrast to TNFR1, which is expressed on a wide range of cell types, DR3 expression is more restricted [6] and most commonly associated with T cells. Two different species of messenger RNA (mRNA) encoding a transmembrane form of DR3, DR3 and DR3β have been isolated from of a panel of human lymphoid cell lines. Eleven splice variants of DR3 mRNA have been identified in peripheral blood lymphocytes, and upon activation by phytohaemagglutinin (PHA), the transcript level is up-regulated [7, 8]. Much less is known about regulation and expression of DR3 protein or about its isoforms in non-lymphoid cells. We have previously reported that DR3 mRNA and protein are induced on non-lymphoid cells of human kidney allografts undergoing acute cellular rejection [9]. Specifically, we observed that DR3 is predominatly up-regulated in tubular epithelial cells but we also observed expression of DR3 in some peritubular endothelial cells (EC), albeit not in glomerular EC.

The only known ligand for DR3 is TNF-like molecule 1A (TL1A), a TNF superfamily member, and mRNA encoding this protein is most abundantly expressed in kidney tissue; it is a long variant of TL1 (also called vascular endothelial growth inhibitor, VEGI) [10]. Both TL1A mRNA and protein are also up-regulated by the same pathological processes that control DR3 expression in renal epithelial cells [11]. In situ hybridization suggested that EC are a major source of TL1A in the human kidney. The TL1A-DR3 system may thus play an important role in the vasculature in renal injury and inflammation. Human umbilical vein (HUV) ECs are a major source of TL1A synthesis among cultured cell types, but it is not known whether EC respond to this ligand. We have investigated fully the expression and function of DR3 in human EC in this study.

## Methods

### Materials

Mouse monoclonal anti-human DR3 was purchased from Chemicon (Southampton, U.K). Rabbit anti-human IκBα and goat anti-human DR3 antibodies were from Santa Cruz (Wiltshire, U.K). Horse anti-goat and goat anti-rabbit horseradish peroxidase (HRP)-conjugated antibodies and Vectashield Mounting Media were from Vector Laboratories Ltd (Peterborough, U.K). Rabbit anti-human NF-κB p65 was purchased from Serotec (Oxford, U.K). Rabbit anti-human phospho-NF-κB p65 was from New England Biolab (Hertfordshire, U.K). Proteinase inhibitor cocktail was purchased from Roche Diagnostics Ltd (East Sussex, U.K). Human recombinant TL1A, human recombinant TNF-α and goat anti-human DR3 antibody were purchased from R&D Systems Europe (Abingdon, U.K). Chicken anti-rabbit Alexafluor^488^, goat anti-mouse Alexafluor^568^, Lipofectin reagent and Opti-MEM media were from Invitrogen Ltd (Paisley, U.K). The ECL system was from Amersham Pharmacia Biotech UK Ltd (Bukinghamshire, U.K).

Unless otherwise indicated, all reagents were from Sigma-Aldrich Company Ltd (Dorset, U.K).

All experiments using human tissue were performed with written, informed consent of patients and the approval of Local Ethical Committee which is East of England - Cambridge Central Research Ethics Committee and Addenbrooke’s Hospital Tissue Bank. Renal tissues was obtained from the uninvolved pole of kidney excised less than half an hour before because of renal tumours or from time zero biopsy of kidney transplant.

### Kidney organ culture

Duplicate 1 mm^3^ fragments of kidney tissue were placed in flat-bottomed 96-well tissue culture plates and immediately immersed in medium M199 containing 10% heat inactivated bovine fetal calf serum (FCS) and 2 mM L-glutamine. Tissue was incubated for 3 hours at 37°C with either culture media alone or with 5 ng/ml TNF-α (TNF) or 0.2 μg/ml TL1A. Half of the harvested tissue was cryoprotected in 30% sucrose in 0.1 M phosphate buffer and snap frozen in isopentane-cooled liquid nitrogen and half was immersed in 4% paraformaldehyde in 0.1 M PIPES buffer pH 7.6 for 1.5 hours at 4°C and processed for paraffin-wax embedding.

### Immunolabelling of tissue

8 μm-thick cryosections of kidney tissue processed as above were permeabilized in cold methanol at -20°C for 5 minutes, washed in Milli-Q water and rinsed in 0.1 M Tris–HCl buffer pH 7.5 containing 0.01% TWEEN-20 (TBS) prior to incubation with blocking buffer (10% FCS in TBS) for 10 minutes. Sections were immunolabelled as previously described [11] Sections were then washed and mounted in Vectashield Mounting Media and imaged with Leica SPE confocal laser scanning microscope (Leica Microsystem Ltd, Milton Keynes, U.K).

### Cell culture

Human umbilical vein EC (HUVEC) were from Lonza (Cambrdige, UK) and serially cultured as previously described [12]. Human dermal microvascular cells (HDMEC) and human pulmonary artery endothelial cells (HPAEC) obtained from Life Technology (Paisley, UK) were cultured following the supplier’s instruction. Cells were used at passages 1–3. Such cultures are free of detectable leukocytes by immunostaining for CD45. TF-1 human erythroleukemia cells obtained from American Tissue Culture Centre (LGC Promochem, Middlesex, U.K) were cultured in media as supplier required.

### RT-PCR

Total RNA was isolated from cells using RNeazy Mini Kit (QIAGEN Ltd, West Sussex, U.K). 1 μg total RNA was amplified with DR3 forward (5′-GTAGCCCCAGGTGTGACTGT-3′) and DR3 reverse (5′-GCTTGAGCATCTCGTACTGC-3′) using Access RT-PCR system (Promega, Southampton, U.K); the PCR product was visualized by 1% low melting temperature agarose gel in Tris-Acetate-EDTA buffer before purified with Wizard PCR Preps (Promega, U.K). The purified DNA was sequenced in Lark Technologies Inc (Essex, U.K).

### Measurement of cell surface receptor expression by flow cytometry

HUVEC were seeded into 6-well tissue culture plate (1.5 × 10^5^ cells per well), and 24 hours later the confluent cells were treated with IFN-γ 500 U/ml, or TNF-α 500U/ml or the combination of both for 24 and 48 hours. Cells were harvested using a non-enzymatic cell suspension solution. TF-1 cells (10^6^/ml) were centrifuged down. Cells were immunolablled and Fixed before analyzed by flow cytometry using FACSCalibur machine (BD Biosciences, Oxford, U.K) as previously described [12]. Data were analyzed using WinMDI 2.8 software.

### Subcellular fractionation

HUVEC were washed once with PBS and scraped into homogenization buffer (10 mM Tris HCl, pH 7.4; 3% sucrose w/v; proteinase inhibitor cocktail, 1 mM PMSF). TF-1 cells were pelleted by centrifugation, washed once with PBS and resuspended in homogenization buffer. All steps were performed at 4°C as previously described [12]. The protein concentration of each fraction was determined by BCA protein assay kit (Pierce, Chester, U.K). Samples were stored at -80°C before analysis by immunoblotting.

### Generation of DR3-flag construct and transfection

The open reading frame of the human DR3 gene was isolated by reverse transcription using the forward primer 5′-TAGCGAATTCAATGGAGCAGCGGCCGCGGG-3′ and the reverse primer 5′-GCGCTCTAGAAACCGTACTTAGGGCTTCTGC-3′. The amplified product was ligated in-frame into the pFlag-CMV1 vector (Sigma-Aldrich Company Ltd, Dorset, U.K). DR3-Flag constructs were introduced into passage 1 HUVEC by transient transfection. In brief, HUVEC grown to 70% confluence on 100 mm diameter plastic culture plates or 6-well plates were transfected approximately 2 hours with DR3-Flag using Lipofectin reagent in Opti-MEM media following manufacture’s instruction. Cells were cultured for another 48 hours. Cells transfected with empty vector or DR3-Flag were treated with TNF 5 ng/ml or TL1A 0.2 μg/ml for 2 or 4 hours. Cells were then collected for analyzing by flow cytometry as described above.

### Immunoblotting

HUVEC transfected with control vector or DR3-Flag were stimulated with 0, 0.02, 0.2, 2 μg/ml TL1A or 0, 1, 5, 10 ng/ml TNF for 30 minutes and then washed once with ice cold PBS before lysed in lysis buffer (62.5 mM Tris, 2% SDS, 10% glycerol, 10 mM sodium orthovanadate, 10 mM sodium fluoride, proteinase inhibitor cocktail). Samples (20 μg for IκBα, 50 μg for subcellular fraction) were immunoblotted and detected as previously described [12]. Serial dilution of samples for immunoblotting confirmed that the density of bands was within the linear range of detection.

### Mouse kidney and heart

Protocols involving animal were approved by the U.K home office and the Cambridge University Local Ethical Committee. C57Bl/6 DR3^-/-^ mice were crossed once into a CD1 background, and the F1 heterozygote progeny were crossed to yield DR3^+/+^ wild type (WT) and DR3^-/-^ knockout (KO) littermates. Animals were killed and the kidneys and hearts were harvested and processed as described in the previous section.

### Statistical analysis

The significance of difference between experimental values was assessed by means of the paired Student’s t test.

## Results

### Activation of NF-κB by TL1A in kidney organ culture

**Figure 1:**
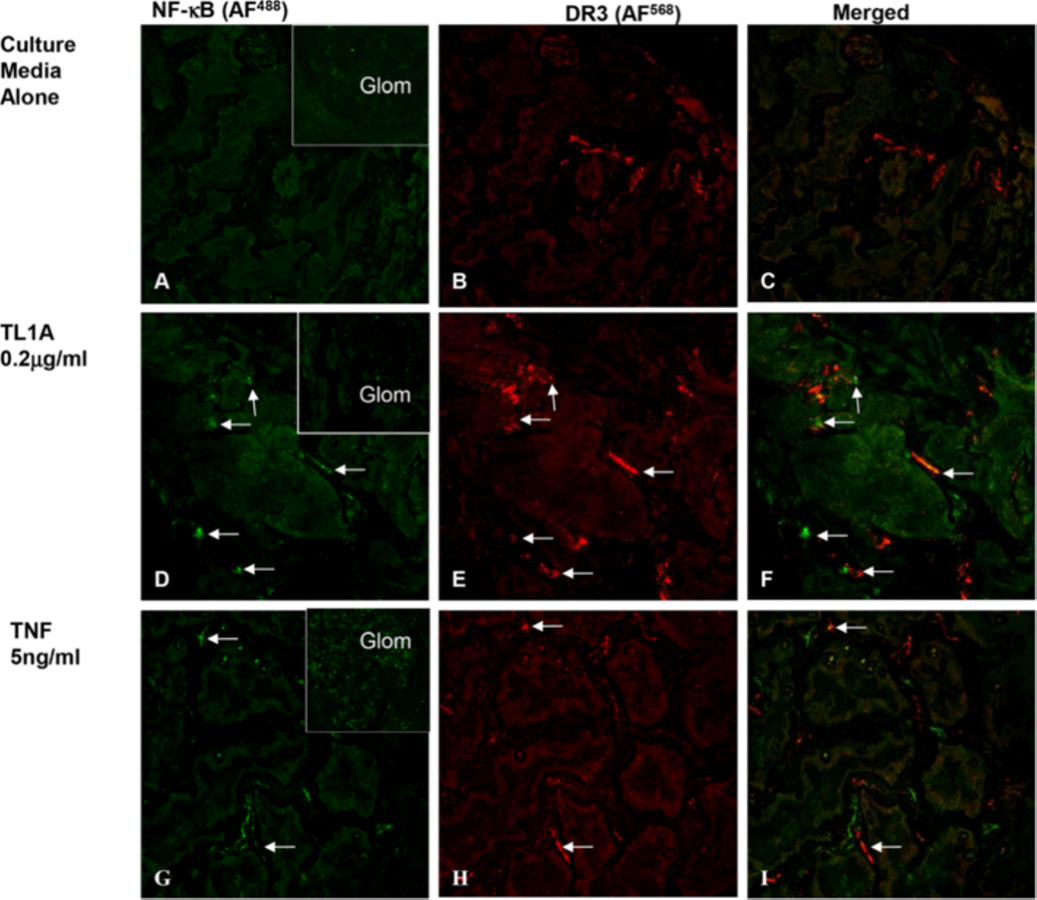
**Response of vascular endothelial cells in human kidney in organ culture to TL1A.** Confocal images of kidney organ culture incubated with either culture media alone or with TL1A (0.2 μg/ml) or TNF (5 ng/ml) for 3 hours at 37°C. **(A-C)** cultures incubated in media alone show EC staining for NF-κBp65 (green) in both the glomerular (inset) and in peritubular vassels. Some peritubular vessels EC showed positive staining for DR3 (red). **(D-F)** Cultures treated with TL1A show co-staining for NF-κBp65 (green) and DR3 (red) in EC of some blood vessel (arrows). Inset; show no signal for NF-κBp65 on glomerular EC. **(G-I)** In contrast, TNF-treated cultures show a strong signal for NF-κB p65 (green) in EC of glomerular (inset) and peritubular blood vessels. DR3 (red) is present only in EC of peritubular blood vessels negative for NF-κB p65. (Original Mags; x40).

We have previously reported that EC in the interstitium of normal human kidney are positive for DR3 but glomerular EC are negative for DR3 [9]. To investigate whether the DR3 positive EC in renal interstitium respond to TL1A, we examined their ability to activate NF-κB in a kidney organ culture model, using TNF treatment as a control. Kidney tissues were treated with media alone, TL1A, or TNF for 3 hours, and then processed and immunostained for the active form of NF-κB (NF-κBp65). No NF-κB activation was detected in untreated normal kidney cultures either in glomerular EC or interstitial EC. Some peritubular capillary EC were positive for DR3 as previously reported (Figure 1A-C). In TL1A-treated cultures, the interstitial EC positive for DR3 showed strong signal for NF-κBp65 indicative of NF-κB activation (Figure 1D-F). NF-κB activation was not observed in glomerular EC (Figure 1D insert). TNF treatment induced more widespread NF-κB activation in interstitial vascular EC and in glomeruli EC (Figure 1G-I), findings which are consistent with our previous report that TNF receptors are widely distributed in kidney tissue.

### Expression of DR3 in cultured EC

**Figure 2:**
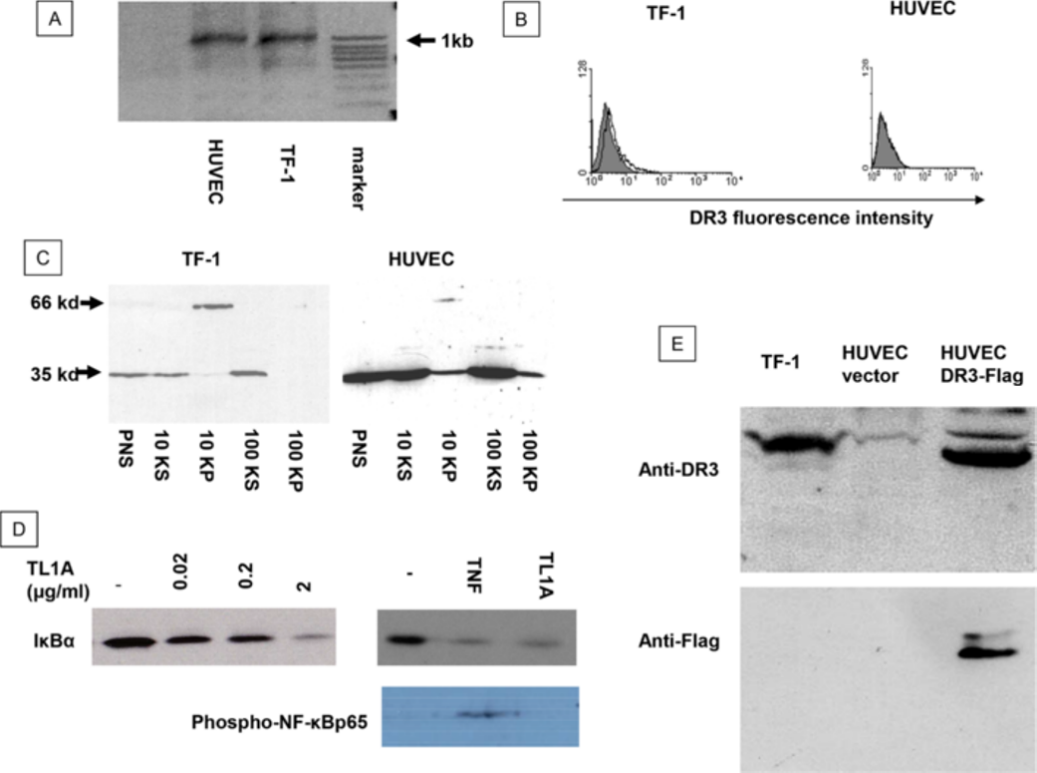
**Expression of DR3 in TF-1 and HUVEC. (A)** RT-PCR showed HUVEC expressed full-length DR3 mRNA, which was the same as that of TF-1 cells. **(B)** Flow cytometry using a monoclonal antibody to DR3 showed positive cell surface staining on TF-1 cells and a very low level of expression on HUVEC. **(C)** Immunoblotting with antibody to DR3 C-terminus showed both cell types express a full-length 59kDa DR3 in the 10KP fraction, containing mainly cell membrane protein. A DR3 band in TF-1 cells was stronger than that of HUVEC with equal protein loading. Both cells also showed a 35kDa band in the soluble subcellular fraction. **(D)** TL1A induced concentration dependent IκBα degradation in TF-1 cells. While TNF (5 ng/ml) and TL1A (0.2 ug/ml) both induced IκBα degradation, phosphor-NFκBp65 was only detected after TNF treatement in the nuclear fraction of TF-1 cells. **(E)** Immunoblot for DR3 in 10K pellet fraction of HUVEC after transfection. Lane 1, TF1 10K pellet as positive control; Lane 2, HUVEC transfected with empty vector; Lane 3, HUVEC transfected with DR3-flag. Two gels with same sample loading were immunoblotted with anti-DR3 antibody (upper blot) and anti-Flag (lower blot). PNS: post nuclear supernatant. 10KS: 10k supernatant. 10KP: 10k pellet. 100KS: 100k supernatant. 100KP: 100k pellet. 50 μg proteins from each fraction was analyzed by immunoblotting, blots were representatives of at least 3 separate experiments.

To investigate mRNA expression for DR3, we extracted total RNA from TF-1 cells (as positive control) and HUVEC. A primer was selected to amplify the full length DR3 open reading frame that extends from exon 2 to exon 10, allowing the possibility of amplifying various splicing variant between exon 3 and 10. A single major PCR product was amplified from HUVEC that was the same size as that of TF-1 cells (Figure 2A), and the sequences of both products were identical (data not shown), indicating that HUVEC express mRNA encoding full length DR3. We then investigated whether HUVEC express DR3 on their surface. Monoclonal anti-DR3 antibody was used to detect cell surface DR3 by flow cytometry. TF-1 cells showed positive fluorescent staining for DR3 (1.61 ± 0.16 units), while there was only weak fluorescent staining for DR3 on HUVEC (0.15 ± 0.01 units) (Figure 2B). To further characterise DR3 protein expression we performed subcellular fractionation and immunoblotting with an anti-DR3 C-terminus antibody. The predicted full length 59 kDa protein was detected in the membrane fraction of both HUVEC and TF-1 cells, with TF-1 expressing more DR3 than HUVEC (Figure 2C), consistent with the results from flow cytometry.

Consistent with previous reports we found that TL1A degraded IκBα in TF1 cells (Figure 2D). However, we did not find evidence of activation of the classical NF-κB pathway in cultured cells. Specifically, treatment with TNF but not TL1A led to p65 phosphorylation in the nuclear fraction of TF1 cells (Figure 2D).

We have found that the magnitude of IκBα degradation following TNF stimulation is related to the number of cell surface TNF receptors [12, 13]. We tried to up-regulate EC’s surface DR3 using cytokines, including TNF-α and IFN-γ alone and in combination without success. Then we transiently transfected HUVEC to increase expression of DR3, using a DR3-Flag construct, followed by flow cytometry and immunoblotting to confirm up-regulation of DR3. Although the major band expressed after transfection was a shorter form of DR3 (as recognized by both anti-DR3 antibody and anti-Flag anti-body), the full length form of DR3 did increase significantly after transfection (Figure 2E).

### Function of DR3 in EC

**Figure 3:**
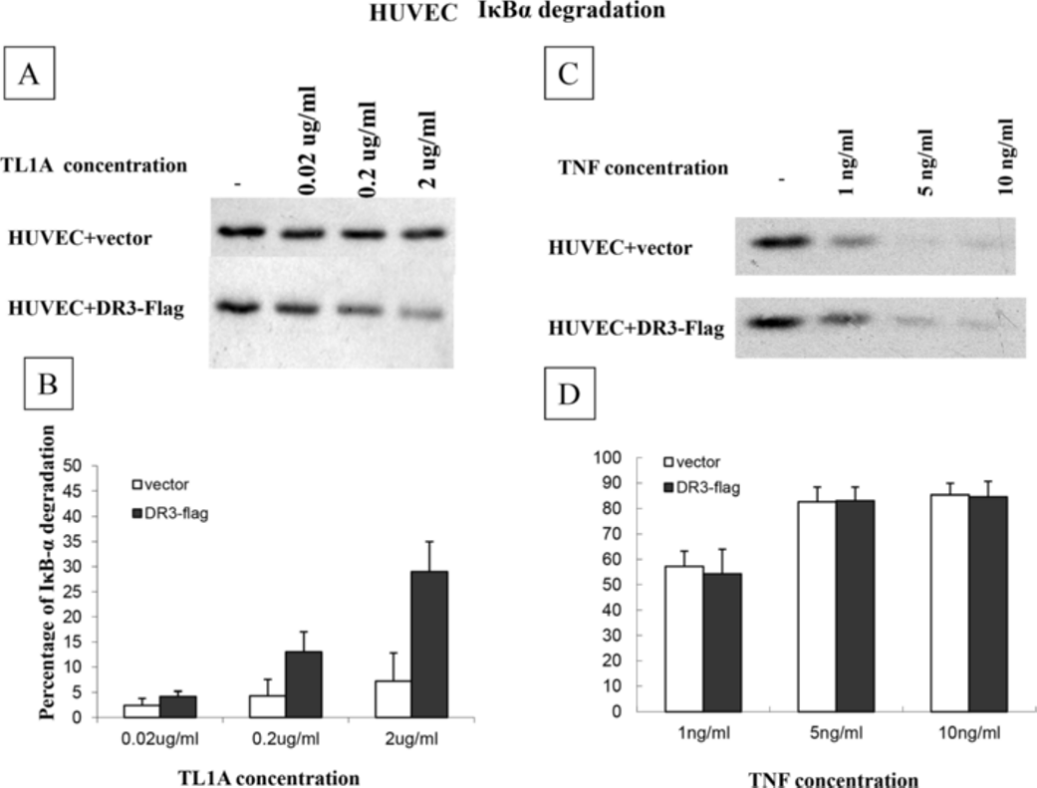
**TL1A induced IκBα degradation in HUVEC. (A)** HUVEC transfected with mock vector or DR3 were treated with ascending concentration of TL1A for 30 minutes, IκBα showed a clear degradation after TL1A stimulation for the DR3-Flag transfected EC. Blots were representatives of 3 separate experiments. **(B)** Densitometry analysis showed a significant increase in IκBα degradation by TL1A at 0.2 and 2 μg/ml for the DR3 transfected EC compared to the mock transfected (13.3 ± 4 vs.4.3 ± 3.2, 29 ± 5.5 vs.7.2 ± 5.6 respectively) (p < 0.05). Values were expressed as percentages of reduction compared to TL1A 0 μg/ml IκBα densities. Results were mean ± SE from 3 experiments. **(C, D)** There was no difference in IκBα degradation after TNF treatment between HUVEC transfected with empty vector and DR3.

DR3 and mock-transfected HUVEC were then stimulated with three different concentrations of TL1A, 0.02, 0.2 and 2μg/ml. Mock-transfected HUVEC treated with 2 μg/ml of TL1A showed some degree of IκB-degradation not statistically significant compared to the untreated cells. In comparison, DR3-transfected HUVEC showed a concentration dependent IκBα degradation (Figure 3A), which was more pronounced after treatment with 0.2 and 2μg/ml TL1A compared to mock-transfected cells (Figure 3A, B). In contrast, there was no noticeable difference in IκBα degradation between mock and DR3-transfected cells after treated with various concentration of TNF (Figure 3C, D).

**Figure 4:**
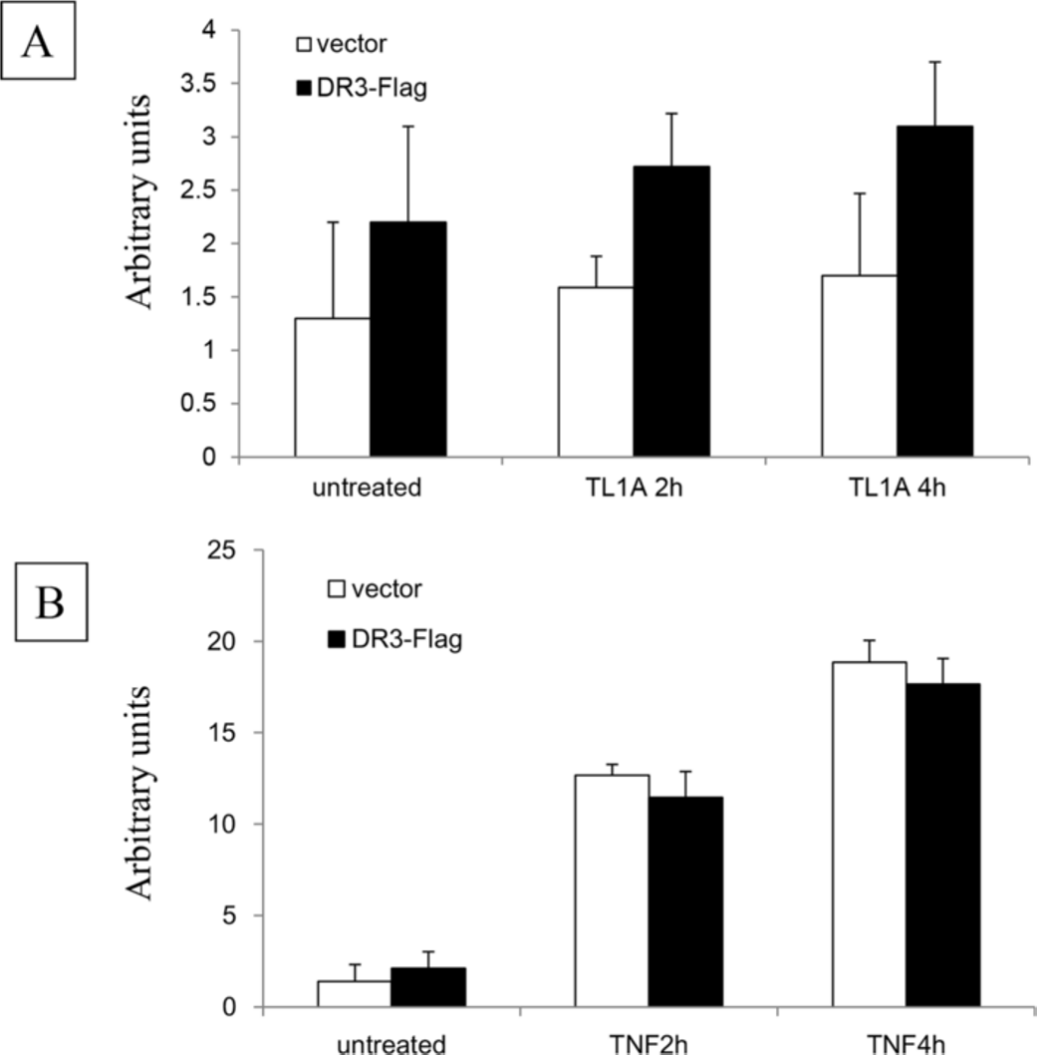
**TL1A induced E-selectin expression in HUVEC.** HUVEC transfected with mock vector or DR3 were treated with 0.2 μg/ml of TL1A for 2 and 4 hours. Cell surface E-selectin was measured by flow cytometry. **(A)** There was no significant change in E-selectin expression for HUVEC transfected with mock vector after TL1A treatment. In contrast, a significant increase in E-selectin expression was detected in HUVEC transfected with DR3 compared with empty vector at each of the two time points measured (1.5 ± 0.2vs.2.7 ± 0.5 and1.7 ± 0.7vs.3.1 ± 0.6) (p < 0.05). There was significant increase in E-selectin expression at 4 hours after TL1A treatment for the HUVEC transfected with DR3 but not at 2 hour time point. Results were mean of units ± SE from 3 experiments. **(B)** There was no difference in E-selectin expression between HUVEC transfected with empty vector and DR3 vector after TNF treatment.

E-selectin expression in HUVEC is NF-κB dependent; we therefore investigated whether the TL1A induced IκBα degradation led to up-regulation of E-selectin. HUVEC transfected with empty vector did not show cell surface E-selectin change after TL1A treatment (Figure 4A). In comparison, transfection with DR3 resulted in a significant increase in expression of cell surface E-selectin after TL1A treatment for 2 or 4 hours compared to the mock transfected. There was up-regulation of E-selectin expression even without TL1A treatment albeit small which might be due to the presence of increased DR3 level and endogenous TL1A in HUVEC. The increased level of E-selectin in DR3-transfected TL1A-treated HUVEC appeared to be time-dependent, significant at a 4 hour time point (Figure 4A). The TL1A effect was slower and less pronounced than that of TNF (Figure 4B), and DR3 transfection had no effect on cell surface E-selectin up-regulation by TNF.

To determine whether EC from other vascular beds constitutively signal through DR3, we studied DR3 response to treatment with TL1A in HDMEC and HPAEC. HDMEC and HPAEC degraded IκBα in response to treatment with TNF, but not TL1A. Consistent with this we could not detect full length DR3 by immunoblotting in HDMEC and HPAEC (see Additional file 1).

### Responses of EC to TL1A in DR3 knockout mouse

**Figure 5:**
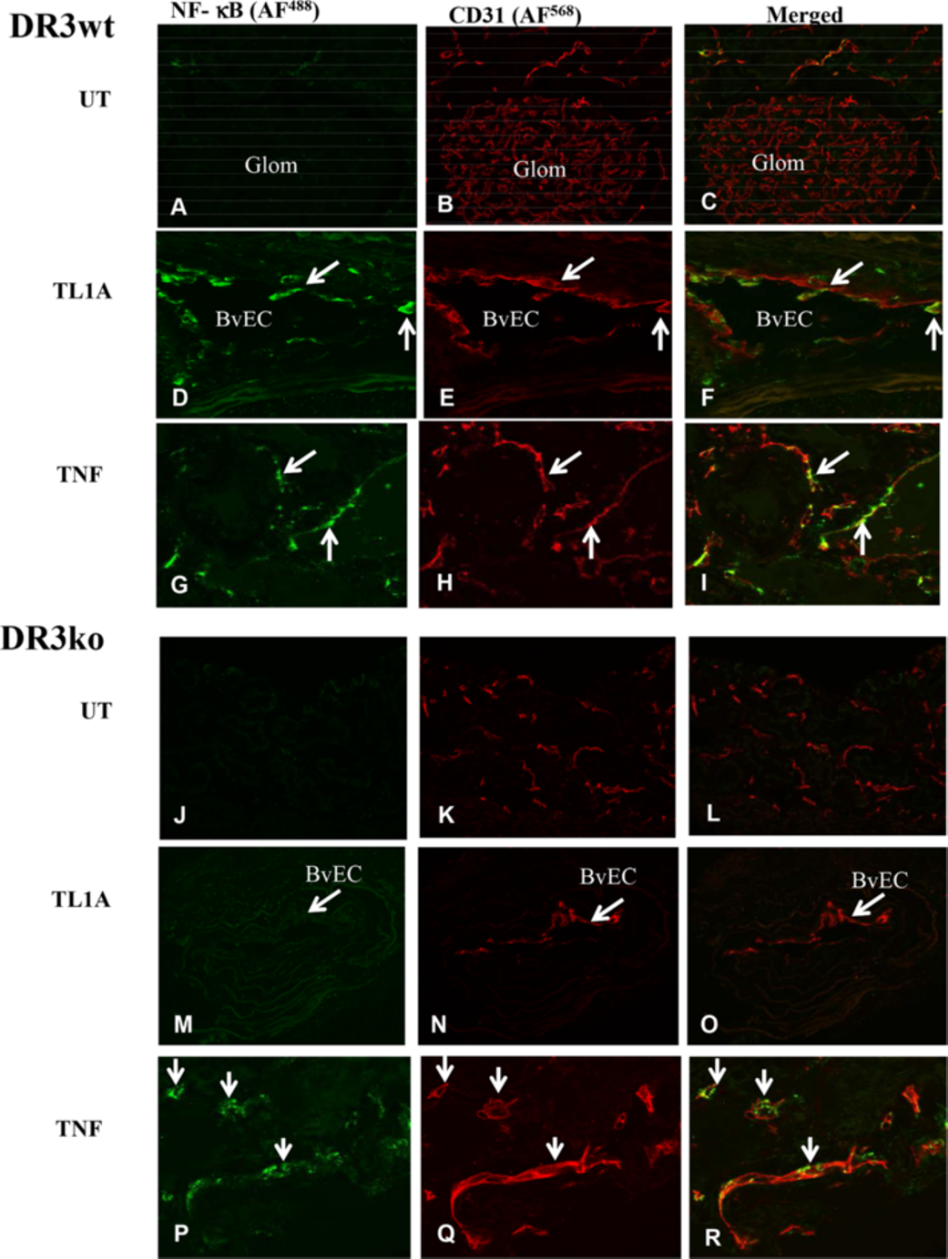
**TL1a induced NF-κB activation in renal interstitial vascular endothelial cells in mouse organ cultures.** Kidney tissue from DR3 wild type (wt) and DR3 knockout (ko) mice were treated with TL1A or TNF and immunolabeled as described in materials and methods. **(A-C)** Untreated (UT) DR3wt showed no positive staining for NF-κB activation in CD31 positive EC from glomerulus or interstitial vessels. **(D-F)** some interstitial EC were positive for NF-κB activation after TL1A treatment. **(G-I)** more vascular EC were positive for NF-κB activation after TNF treatment. **(J-L)** untreated DR3ko showed no staining for NF-κB activation in vascular EC. **(M-O)** TL1A-treated DR3ko showed no staining for NF-κB activation in vascular EC. **(P-R)** TNF-treated DR3ko showed positive staining for NF-κB activation in vascular EC. Arrow for BvEC: blood vessel endothelial cells. Results were representative of 3 experiments.

**Figure 6:**
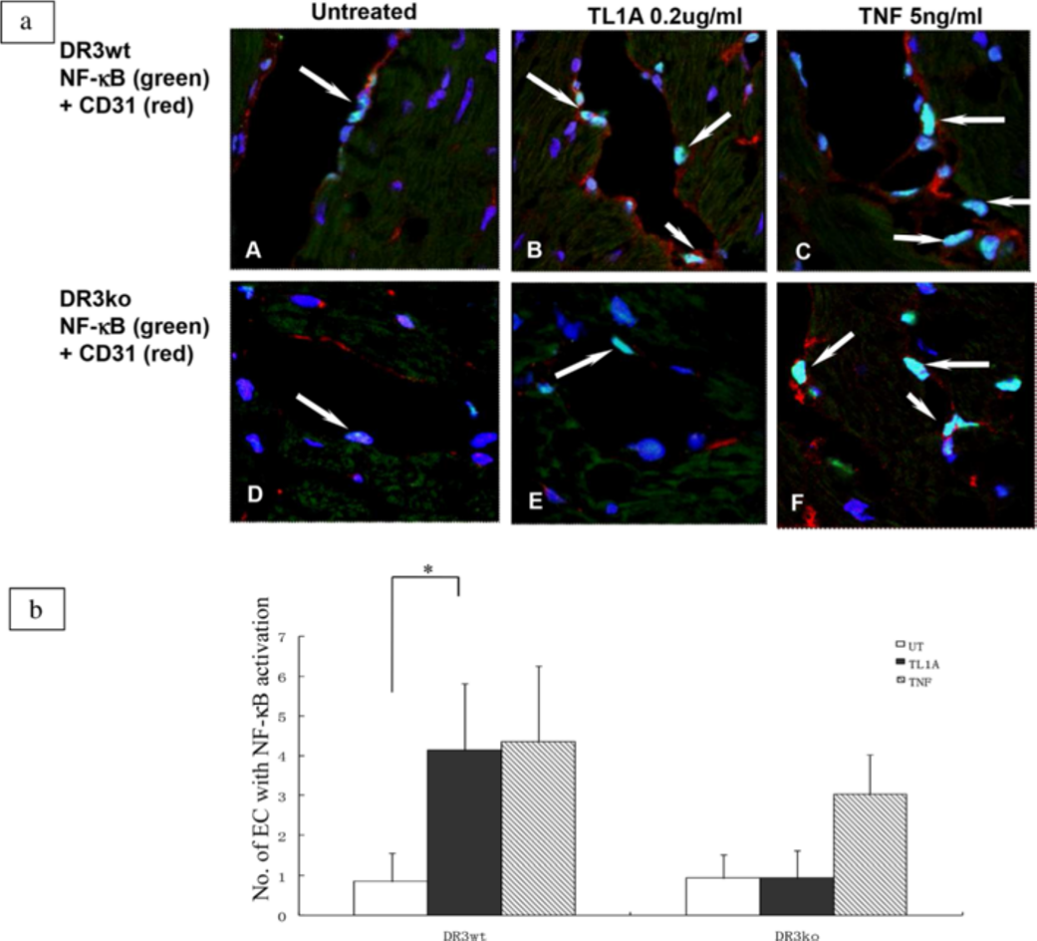
**TL1A induced NF-κB activation in vascular endothelial cells in mouse heart organ cultures. (6a)** Heart tissue from DR3 wild type (wt) and DR3 knock-out (ko) mice were treated with TL1A or TNF and immunolabeled as described in materials and methods. DR3wt showed more nuclear NF-κB activation in CD31 positive EC after TL1A treatment **(B)** compared to the untreated **(A)**. There was similar odd nuclear NF-κB activation in DR3ko mice EC between TL1A treated **(E)** and untreated cultures **(D)**. TNF induced a more profound nuclear NF-κB activation in EC of both DR3wt and DR3ko **(C** and **F)**. Results were representative of 3 experiments. **(6b)** Graph showed a statistical difference in number of EC in TL1A treated tissue with NF-κB activation in DR3wt compared to DR3ko (p < 0.05) (*).

We next examined the effects of TL1A on DR3 knockout (KO) mice to verify the specificity of in vitro data. Untreated cultures of DR3 wild type (WT) and DR3ko mouse showed no EC positive for NF-κB/p65 (Figure 5A-C and J-L). DR3wt but not DR3ko cultures treated with TL1A showed NF-κB activation in EC (Figure 5D-F and M-O), while TNF treatment induced a similar degree of NF-κB activation in EC in both DR3wt and DR3ko cultures (Figure 5G-I and P-R). To determine whether TL1A can activate EC in other vascular beds we examined the effects of TL1A on mouse heart tissue. There were occasional EC positive for NF-κB/p65 in untreated tissue of DR3wt and DR3ko mice (Figure 6a A, D), TL1A treatment induced NF-κB activation in EC in DR3wt mice but not DR3ko mice (Figure 6a B, E and Figure 6b), while TNF induced a similar degree of NF-κB activation in EC in DR3wt and DR3ko mice (Figure 6a C, F and Figure 6b).

## Discussion

In this study we have shown for the first time that human EC express full length DR3, which supports a more widespread cellular expression of DR3 than its original description as a lymphoid cell receptor [14–16]. Moreover, EC have all the machinery necessary to respond upon DR3 up-regulation. Exogenous TL1A can induce NF-κB activation in EC both in vitro and in vivo.

TNF superfamily members play an important role in renal injury [17], and the differential expression of TNF receptor (TNFR) superfamily members may have functional implications [18]. TNFRs are widely expressed in glomerular and interstitial EC, while DR3 is detected only in some interstitial EC. Our results show that, like TF-1 cells, HUVEC express both DR3 transcript and protein. Although we were unable to identify a pathophysiological stimulus that would up-regulate full length DR3 in EC, we were able to increase surface expression of DR3 by transfection, which resulted in IκBα degradation by TL1A treatment. This potential function of DR3 in EC is supported by our in situ study of EC in TL1A-treated kidney tissue.

Pre-ligand-binding assembly of TNFR into trimer complexes is critical in TNF mediated signaling [19], which is based on a ligand-receptor trimerisation. It is possible that TL1A signaling does not occur in untreated HUVEC because there is both insufficient level of DR3 on the surface, and other forms of non-signaling DR3 interfere with TL1A binding. The lack of response to TL1A cannot be attributed to inability of DR3 to signal IκBα degradation in HUVEC, as expressing full length DR3 by transfection allowed TL1A to degrade IκBα. The lower molecular weight band detected by immunoblotting with anti-flag and anti-DR3 after transfection may be an isoform of DR3 that is able to bind TL1A and interfere with its signalling, as reported for other death receptors like DR4 and DR5 [20]. This DR3 isoform differs from the reported soluble form of DR3 [9] as it is detected only in the membrane fraction and not the soluble fraction. It is unlikely to be the signaling isoform inducing IκBα degradation as it differs from the isoform expressed in TF-1 cells. Moreover, the relatively abundant expression of this shorter isoform after transfection did not make HUVEC more responsive to TL1A than TF-1 cell, which in turn indicate that it is full-length DR3 that TL1A signals through. Further studies on isoforms of DR3 are needed to support our hypothesis. Our results are consistent with Migone et al., when they over expressed TL1A and DR3 in 293T cells, NF-κB activation could be detected, whereas VEGI failed to do so [10].

NF-κB factors belong to the Rel family of transcription factor; they mediated biological activity through classical and alternative pathways. Our results in tissue support DR3 signaling through the classical pathway similar to TNFR1, which induces IκBα degradation and RelA/p65 activation [17]. Although we were unable to demonstrate activation of RelA/p65 in TF1 cells following IκBα degradation, we have shown that TL1A induces RelA/p65 activation in EC in situ. Furthermore, we demonstrated TL1A induced E-selectin expression in DR3 transfected HUVEC, indicating functional activation of NF-κB in these cells. We have previously observed differences in death receptor signaling in situ compared to in vitro [18]. Whether the alternative pathway is involved requires further studies. Although TL1A-DR3 interaction has been extensively studied in autoimmune diseases such as inflammatory bowel diseases, experimental allergic encephalomyelitis and rheumatoid arthritis [21–23], its physiological role in immune reaction is still unclear. Furthermore it has been shown to have functions outside of the immune system, including atherosclerosis and aging [24, 25]. Both TL1A and DR3 expression levels are low under physiological conditions, but can be acutely up-regulated in inflammatory reaction. As EC also express TL1A, the dynamic balance of the ligand and receptor in endothelial systems could be critical for maintaining normal function of these cells. Over expression of either ligand or receptor could lead to inflammation or apoptosis [26]. In normal kidney where most cells are negative for DR3, TL1A may be unable to induce signaling. However, in human allograft rejection infiltrating mononuclear cells, which express high levels of DR3, are able to respond to TL1A, the DR3-TL1A interaction may form a positive feedback loop inducing an inflammatory reaction.

## Conclusion

EC express full length and functional DR3. The TL1A and DR3 system may play an important role in vascular injury in allograft rejection and atherosclerosis where significant increases in both TL1A and DR3 have been reported.

## Electronic supplementary material

Additional file 1: **Expression and signaling of DR3 in human microvascular and pulmonary artery endothelial cells.** **(A)** Whole cell lysates from HMVEC and HPAEC were immunoblotted for DR3 expression. TF-1 was positive for full length DR3, while both endothelial cells were negative. **(B)** HMVEC and HPVEC were treated with ascending concentration of TL1A, there was no IκBα degradation detected. (TIFF 292 KB)
