# Supplementary figures and images for: TL1-A can engage death receptor-3 and activate NF-kappa B in endothelial cells
Source: BMC Nephrol. 2014 Nov 16;15:178. doi: 10.1186/1471-2369-15-178 (PMC4239315; doi:10.1186/1471-2369-15-178)

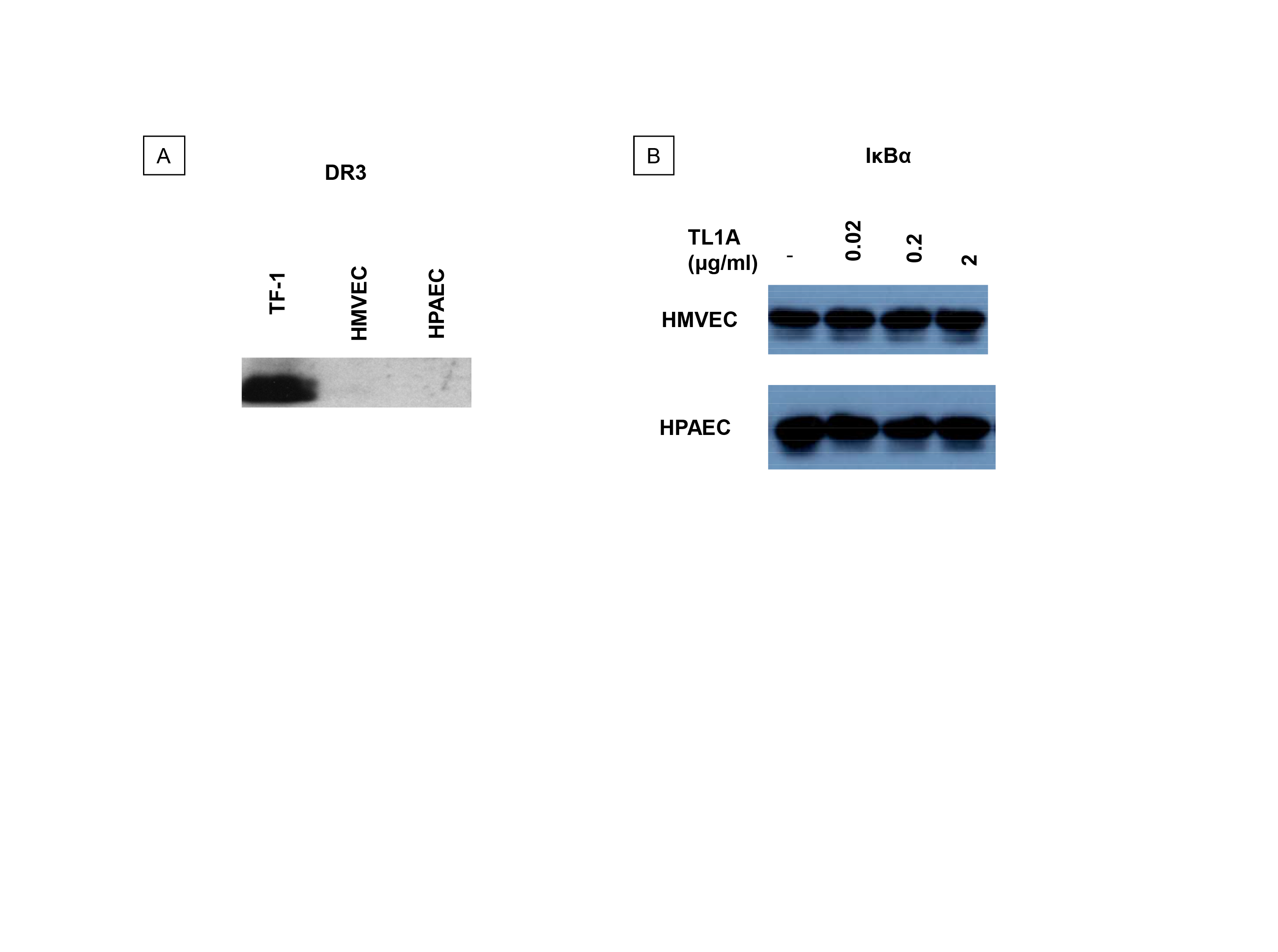

Supplement: Supplementary file 1 — Additional file 1: Expression and signaling of DR3 in human microvascular and pulmonary artery endothelial cells. (A) Whole cell lysates from HMVEC and HPAEC were immunoblotted for DR3 expression. TF-1 was positive for full length DR3, while both endothelial cells were negative. (B) HMVEC and HPVEC were treated with ascending concentration of TL1A, there was no IκBα degradation detected. (TIFF 292 KB) [file 12882_2014_866_MOESM1_ESM.tiff]
